# Death

**Published:** 1887-04-30

**Authors:** 


### Words of Consolation.

[Communications for this column should be addressed, "Chaplain," care of the Editor of The Hospital, who will gratefully welcome any suggestions or inquiries from matrons, nurses, and the staff generally.]

### XXXI.—Death.

God forbid that anything we have written upon this solemn subject should be the means of dispelling the fear of death from hearts that still ought to retain it. The difficulty is when people have long deceived themselves in sin, for them to become sufficiently inspired with such fear for it to lead to true repentance. Sickness will quicken the fear in many cases which has been dormant, though never quite absent, for years and years; and so sufferers find, instead of the occasional pricks of conscience experienced during health, that a horrible dread hath overwhelmed them, as the terrors of death lay hold upon them. We are mistaken if we ever attempt to minister comfort in such cases, unless some decided sorrow for the past is expressed. The comfort (if so called) must rather take the shape of assurances that the horrible dread is permitted in God's mercy in the hope of its becoming a stepping-stone to love. "Perfect love casteth out fear" (1 John iv, 18). But in those who have never learnt to love, or have ceased to love, fear, if not too soon smothered by false prophets, or unconditional promises of peace, may have a most wholesome effect. It is quite possible to drive away fear before any real convictions of sin have been given a place in the heart; and then follows that fatally stolid, petrified indifference to all religious impressions which seems to end for this present time in sleep, long before death. Of such, alas! it is but too true, "They are not only asleep, but dying."

But passing once more from the spiritually dead to others whose conversion has been real, or who may never have fallen far from Baptismal grace, we ask, What is the Christian religion worth in each of our cases; what is the value of our faith; even if it has done much to alter our views of life, but little to alter our views of death? "Yea, though I walk through the valley of the shadow of death, I will fear no evil: for thou art with me; thy rod and thy staff comfort me" (Psalm xxiii. 4). If in spite of such comfortable words, supported by the assurances of the New Testament, we any of us allow a dominion to fears which we might abolish altogether by living in a higher and better faith, the fault will be our own if death at last proves to be itself, and not the blessed sleep the Gospel takes if for. For Christians to indulge any morbid dread under the tenderest care of the Good Shepherd, in the very arms of the Lord of Life, who now holds the keys of death and of hell, is to doubt His love, who keepeth Israel and will not suffer the sun to burn them by day, neither the moon by night (Psalm cxxi).

"As a child, when wearied with play, nestles in his mother's arms, and, without one lingering fear of danger, sweetly sleeps, so does the weary believer, when called to die, nestle with fearless confidence in the Everlasting Arms. What mother-love is to the confiding child, the all-embracing love of the Infinite One is to the dying Christian man whose faith recognises the Father in the gracious face of the glorified Jesus. It was because Stephen's eye was fixed on that Divine face, while he lay bleeding on the rough bed of martyrdom, that inspiration did not say he died—even that was too harsh a term—but that he 'fell asleep.' O beautiful conception of death! Going into a soft, sweet sleep, which ends the sorrow and toils of earth, and is followed by a waking amidst the music, the bliss, of Paradise, and a beholding of the beauty and love of God in the face of Him who is the altogether lovely, the Son of Man, the Son of God—this is death." (*Great Thoughts.*)

(*To be continued.*)

